# Curcumin/Carrier Coprecipitation by Supercritical Antisolvent Route

**DOI:** 10.3390/pharmaceutics16030352

**Published:** 2024-03-02

**Authors:** Stefania Mottola, Iolanda De Marco

**Affiliations:** 1 Department of Industrial Engineering, University of Salerno, Via Giovanni Paolo II 132, 84084 Fisciano, Salerno, Italy; smottola@unisa.it; 2 Research Centre for Biomaterials BIONAM, University of Salerno, Via Giovanni Paolo II 132, 84084 Fisciano, Salerno, Italy

**Keywords:** inclusion complexes, coprecipitated microparticles, β-cyclodextrin, SAS precipitation, fast release, supercritical CO_2_

## Abstract

In this work, polyvinylpyrrolidone (PVP)- and β-cyclodextrin (β-CD)-based composite powders containing curcumin (CURC) were obtained through the supercritical antisolvent (SAS) technique. Pressure, total concentration of CURC/carrier in dimethylsulfoxide, and CURC/carrier ratio effects on the morphology and size of the precipitated powders were investigated. Using PVP as the carrier, spherical particles with a mean diameter of 1.72 μm were obtained at 12.0 MPa, 20 mg/mL, and a CURC/PVP molar ratio equal to 1/2 mol/mol; using β-CD as the carrier, the optimal operating conditions were 9.0 MPa and 200 mg/mL; well-defined micrometric particles with mean diameters equal to 2.98 and 3.69 μm were obtained at molar ratios of 1/2 and 1/1 mol/mol, respectively. FT-IR spectra of CURC/ β-CD inclusion complexes and coprecipitated CURC/PVP powders revealed the presence of some peaks of the active compounds. The stoichiometry of the complexes evaluated through the Job method revealed that β-CD formed inclusion complexes with CURC at a molar ratio equal to 1/1. Dissolution profiles revealed that in comparison with the curve of the pure ingredient, the SAS-processed powders obtained using both PVP and β-CD have an improved release rate.

## 1. Introduction

In recent decades, research has increasingly focused on the importance of compounds of natural origin that have protective effects against cellular oxidation [1,2]. They act as antioxidants in the human body by dropping harmful free radicals; therefore, they can prevent or alleviate many diseases. Most of these compounds are polyphenols, plant compounds naturally present in plant foods, such as vegetables, fruit, dark chocolate, and wine. Polyphenols are in some cases pigmented (such as anthocyanins or curcuminoids), in others colorless (such as flavonols or isoflavones), and have antioxidant, anti-inflammatory, antibacterial, and antitumor properties [3]. Polyphenols have become key elements in scientific research: they have stimulating biological activities, and they act as chelators in the capture of free radicals, which are responsible for many diseases, including Parkinson’s disease, Alzheimer’s disease, diabetes mellitus, and cardiovascular diseases. In addition, polyphenols protect against damages caused by UV rays and viral, fungal, and bacterial infections; inhibitory effects have also been reported at distinct stages of cancer development [4]. Research focusing on the anticancer properties of polyphenols is of particular importance because of the limitations of current anticancer therapies, such as cell sensitivity to irradiation, incomplete tumor resection, or resistance to chemotherapy drugs. To overcome the shortcomings of current methodologies, scientific research is focusing on developing new anticancer agents with greater effectiveness and reduced side effects. Phytotherapy, among others, has proved to be a potential alternative that exploits the therapeutic properties of plants to produce drugs to treat several types of cancer. Although the mechanisms of action of these molecules are still largely unknown, research into the anticancer effects of plant compounds is increasing [5,6,7,8,9,10]. 

Among the different polyphenols, curcumin (CURC) [1,7-bis(4-hydroxy-3-methoxyphenyl)-1,6-heptadiene-3,5-dione] is the main constituent of the rhizomes of the plant *Curcuma longa* and is the substance responsible for the yellow color of turmeric. It is a potent antioxidant, anti-carcinogenic, anti-inflammatory, anti-angiogenic, antispasmodic, antimicrobial, and anti-parasitic ingredient [11,12]. In the last twenty years, research has been interested in this compound for its potent antiamyloidogenic effects in the treatment of Alzheimer’s disease [13]. Although it is a natural compound and therefore has less toxicity when compared with other active principle ingredients (APIs), this compound must often be administered at high concentrations due to its poor bioavailability, resulting from a low solubility in water [14,15]. To avoid the administration of considerable amounts of the API, it is often proposed as micronized powders [16,17]; moreover, to protect it from light and oxygen, it is often coprecipitated with a hydrophilic carrier [18,19]. Among the innovative techniques used in the pharmaceutical field to obtain carrier/active principle composites, the ones based on supercritical carbon dioxide (scCO_2_) are particularly attractive [20,21,22]. Indeed, fluids at supercritical conditions have peculiar characteristics, intermediate between the ones of a gas (like diffusivity) and a liquid (e.g., density) [23,24,25]. Among the supercritical fluids-based processes used to micronize and/or coprecipitate APIs, the supercritical antisolvent (SAS) precipitation is particularly used as it exploits the solubility of many APIs in organic solvents and the miscibility of these solvents in scCO_2_ under process conditions. Another important prerequisite for the success of the process is that the API is insoluble, at the process conditions, in the mixture made up of the organic solvent and scCO_2_, which therefore has the role of antisolvent. The main advantage of using carbon dioxide with the role of an antisolvent is that it completely removes the solvent, avoiding further processes to eliminate any traces of it. Among the different carriers used to coprecipitate the principles of interest, satisfactory results have been obtained using polyvinylpyrrolidone (PVP) [26,27] or β-cyclodextrin (β-CD) [28,29,30]. PVP is one of the most used vinyl polymers in the pharmaceutical and biomedical sectors due to its interesting properties, such as good stability, bio- and hemocompatibility, biodegradability, and extremely low cytotoxicity. This polymer is used in a variety of applications and is approved by the Food and Drug Administration as a safe polymer for pharmaceutical applications. Low-molecular-weight PVP has been shown to be completely eliminated via the kidneys after oral administration [31]. Cyclodextrins are cyclic oligosaccharides derived from starch, containing some α-D-glucopyranose units (seven in the case of β-CD), characterized by a hydrophilic truncated cone shape exterior and a hydrophobic conical cavity [32]. The API is uniformly dispersed into the carrier matrix in the former case, while inclusion complexes are obtained in the latter case.

To improve the bioavailability of curcumin, in this paper, the SAS process has been employed to obtain β-CD-based and PVP-based microparticles. The reason for processing CURC using a carrier is the increased API’s dissolution rate in water, making it an excellent candidate for preventing/treating many diseases adjuvant to conventional treatments. The effect of different operating parameters on the morphology and dimensions of the powders will be evaluated to find the most suitable process conditions.

## 2. Materials and Methods

### 2.1. Materials

Materials used for the experimentation are CO_2_ (purity 99%) bought by Morlando Group S.p.A. (Sant’Antimo, Italy); dimethylsulfoxide (DMSO, purity 99.5%) provided by Carlo Erba (Cornaredo, Italy); β-cyclodextrin (β-CD, purity 98%) supplied by Acros Organics; and polyvinylpyrrolidone (PVP, molecular weight 10 kg/mol) and curcumin purchased from Aldrich Group (Milan, Italy). 

### 2.2. Apparatus and Procedure

**Figure 1:**
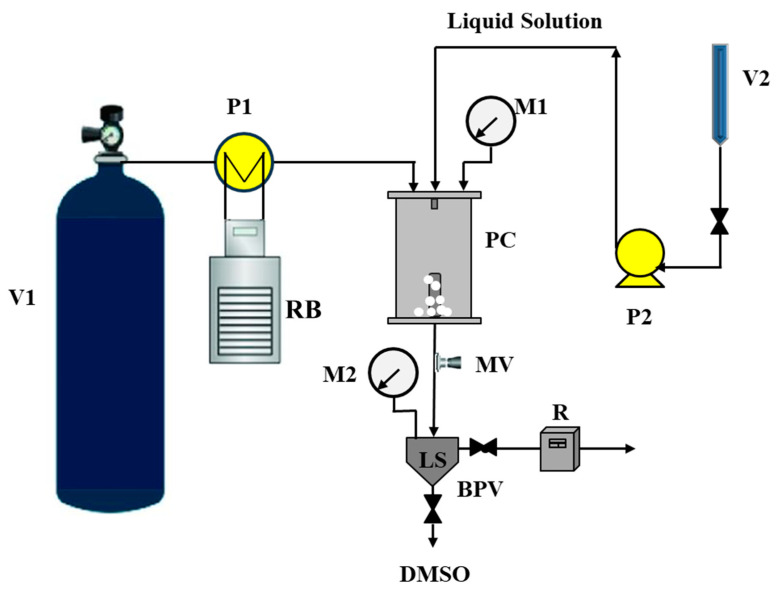
Scheme of SAS plant. BPV: back-pressure valve; LS: liquid separator; M1, M2: manometers; MV: micrometric valve; P1, P2: pumps; PC: precipitation chamber; R: rotameter; RB: refrigerating bath; V1: CO_2_ container; V2: liquid solution burette.

Figure 1 depicts the laboratory SAS plant, which includes a precipitation chamber (PC) with an inside volume of 500 cm^3^. CO_2_, which acts as an antisolvent, is supplied to the chamber from tank V1 via a pump (P1). The liquid solution is stored in a burette (V2) and injected into PC using a stainless-steel nozzle by means of the pump P2. Before entering the chamber, the CO_2_ is chilled in a refrigerating bath (RB). The pressure inside the PC is adjusted by a micrometric valve (MV) and checked using a manometer (M). A proportional–integral–derivative (PID) controller is connected to heating bands to maintain the desired operating temperature. A steel filter is positioned at the base of the PC and gathers the precipitated powder while enabling the CO_2_-DMSO mixture to flow through. DMSO is then reclaimed in a liquid separator (LS). The pressure within LS is controlled by a back-pressure valve (BPV). The flow rate is monitored by a rotameter (R).

In short, a SAS experiment begins stabilizing the desired pressure and temperature after pumping CO_2_ into the vessel. Then, the liquid solution containing the solutes is sprayed into the PC. Subsequently, the solvent residues are eliminated thanks to the CO_2_, which continues to flow. Next, the CO_2_ pump is switched off, the precipitator is decompressed to atmospheric pressure, and the precipitated powders are collected and characterized [33]. 

### 2.3. Analyses

#### 2.3.1. Morphological Characterization and PSD Evaluation

The morphology of the powders was examined via a field emission scanning electron microscope (FESEM, model LEO 1525, Carl Zeiss SMT AG, Oberkochen, Germany). After each experiment, the collected powder was spread on a carbon tab and covered with a gold–palladium layer (thickness 250 Å). 

Particle size distributions (PSDs) were obtained through image analysis thanks to the Sigma Scan Pro 5.0.0 (Aspire Software International, Ashburn, VA, USA) software, which allows the measurement of the particle diameters, and Microcal Origin Pro 9.8 (OriginLab Corporation, Northampton, MA, USA), which calculates the size distributions and provides the statistical parameters of themselves. When nanometric powders were precipitated, the PSDs were obtained via dynamic light scattering (DLS) with a Zetasizer (mod. 5000, Malvern Instruments Ltd., Worcestershire, UK). In particular, a suspension was prepared by placing the coprecipitated powders in a glass cuvette with ethyl acetate in the case of cyclodextrin-based inclusion complexes and acetone in the case of PVP-based composite microspheres. The suspension was stabilized, adding a minimum amount of surfactant, which was Span80 for cyclodextrin-based complexes and Tween 80 for PVP-based coprecipitates.

#### 2.3.2. FT-IR Analysis

Fourier transform infrared (FT-IR) spectra were assessed using an IRTracer-100 (SHIMADZU Europe, Duisburg, Germany) with a resolution of 0.5 cm^−1^. A total of 1 mg of sample was mixed with 100 mg of KBr powder to make the discs transparent to infrared. The analysis was then carried out in a scan wavenumber range of 4000 to 500 cm^−1^. 

#### 2.3.3. Dissolution Tests and Encapsulation Yield

Dissolution tests were conducted with a Cary 60 (Varian, Palo Alto, CA, USA) UV/vis spectrophotometer. SAS sample containing 5 mg of CURC was suspended in 3 mL of pH 7.4 PBS and placed inside a dialysis sack to simulate the physiological conditions. The sack was immersed in 300 mL of pH 7.4 PBS, stirred constantly at 150 rpm, and maintained at a temperature of 37 °C.

Once the plateau is reached, the release test is conducted, with the understanding that all drugs have moved to the outer phase. The analyses were carried out in triplicate to guarantee the repeatability of the results.

Absorbance measurements were taken using UV-vis analysis after the release to find the encapsulation yield. Loading efficiency was calculated using the following formula:

(1) $$\mathrm{EE}\%=\frac{\mathrm{Abs}\;\mathrm{measured}}{\mathrm{Abs}\;\mathrm{theory}}\times 100$$

#### 2.3.4. Job Method

The stoichiometry of the CURC/β-CD inclusion complexes was estimated using Job plot. Two solutions with equimolecular concentrations of CURC and β-CD in distilled water were prepared. Subsequently, different volumes of said solutions were mixed to have a constant total concentration and mole fractions of X = [CURC]/([CURC] + [β-CD]) varying in the range 0–1. The samples were sonicated for 15 min and stirred for 72 h at room temperature to be sure that the equilibrium had been reached. After a dilution, the solutions were analyzed by UV/vis spectroscopy at a wavelength of 425 nm. Job plots are, then, built by plotting ΔA × X versus X, where ΔA is the difference in absorbance without and with β-CD, and X is the molar ratio CURC/β-CD. The maximum of the curve figures out the stoichiometry of the inclusion complex.

## 3. Results and Discussion

In this paper, the SAS technique was employed to process curcumin to obtain both coprecipitated powders (PVP as the carrier) and inclusion complexes (using β-CD). The presence of hydrophilic carriers helps improve the bioavailability of the active principles thanks to an increase in the dissolution rate. The results obtained previously with the SAS process indicate that, among the various potential hydrophilic carriers, these are the ones that best allow obtaining micrometric-sized particles with significantly faster release profiles of the active compound [20]. All tests were conducted with an injector diameter of 100 μm and using DMSO as the solvent because it has been previously demonstrated that microparticles are formed with the SAS process when solvents, like DMSO, with a large transition pressure range from the two-phase to the one phase mixing behavior are used [34]. Furthermore, the following operating conditions were set and chosen based on results previously obtained: carbon dioxide flow rate of 16,667 mL/min; solution flow rate equal to 1 mL/min; temperature fixed at 40 °C. The antioxidant was processed with the two carriers varying different operating conditions, such as total concentration (C_tot_) of the solutes (CURC and carrier) in DMSO, pressure (P), and CURC/carrier ratio, as reported in Table 1. In addition of the operating conditions, the main results in terms of morphology of the precipitated powders, mean diameter of the particles (m.d.), standard deviation (s.d.) of the PSD on a volumetric basis, and encapsulation efficiency (EE%) are also indicated in Table 1.

Before carrying out the coprecipitation and complexation experiments, a SAS test was performed using the active ingredient alone without carriers. Experiment #1 was conducted at P = 9.0 MPa and C_tot_ = 20 mg/mL. When opening the precipitation vessel, no powder was precipitated, indicating that the solute was soluble in correspondence with the chosen process conditions in the mixture of scCO_2_ and DMSO. This preliminary experiment proved that the presence of a carrier is necessary to process curcumin using the SAS technique.

### 3.1. Experiments with β-Cyclodextrin

The SAS precipitation of CURC was then attempted using β-CD as the carrier. In the following sub-sections, the effect of the total concentration, pressure, and API/carrier ratio was investigated, and the results were presented and discussed.

#### 3.1.1. Effect of the Concentration

**Figure 2:**
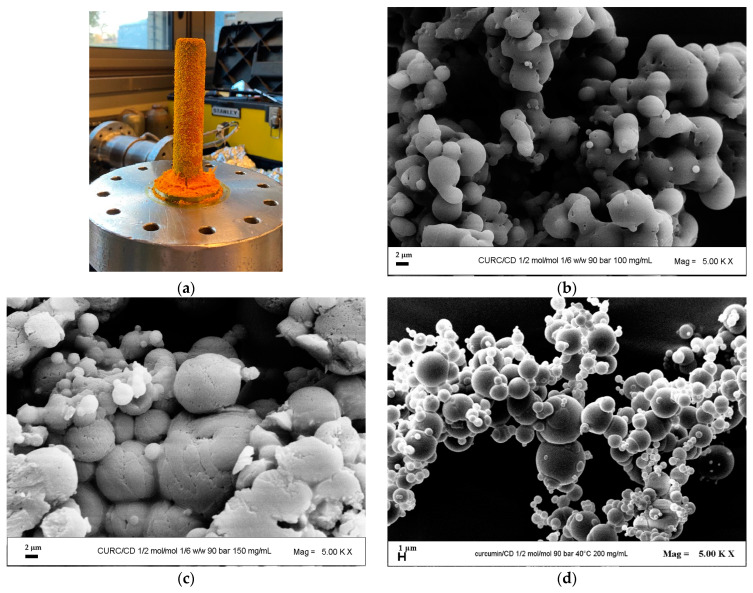
SAS powders of CURC/β-CD processed at 9.0 MPa and 1/2 mol/mol. (**a**) frame of the filter at the opening of the precipitator; FESEM images at (**b**) 100 mg/mL, (**c**) 150 mg/mL, and (**d**) 200 mg/mL.

The effect of the total concentration was evaluated at P = 9.0 MPa and a curcumin/β-CD ratio equal to 1/2 mol/mol. Unlike when processing the active compound alone, the presence of the carrier allowed the attainment of powder at the opening of the precipitator, as can be seen from the photo shown in Figure 2a, reporting the filter covered with powder of an orange color typical of curcumin. As can be observed by the FESEM images, the increase in C_tot_ from 100 mg/mL to 200 mg/mL led to an evident variation in the morphology. Indeed, at 100 mg/mL (FESEM image reported in Figure 2b), the powder was constituted by coalescing particles; at 150 mg/mL (Figure 2c), there is an improvement in terms of sphericity of the particles which, however, are still slightly aggregated; only by setting the concentration at 200 mg/mL were very distinct microparticles obtained (Figure 2d).

The following experiments were performed at a total concentration of 200 mg/mL due to the results described.

#### 3.1.2. Effect of Pressure

**Figure 3:**
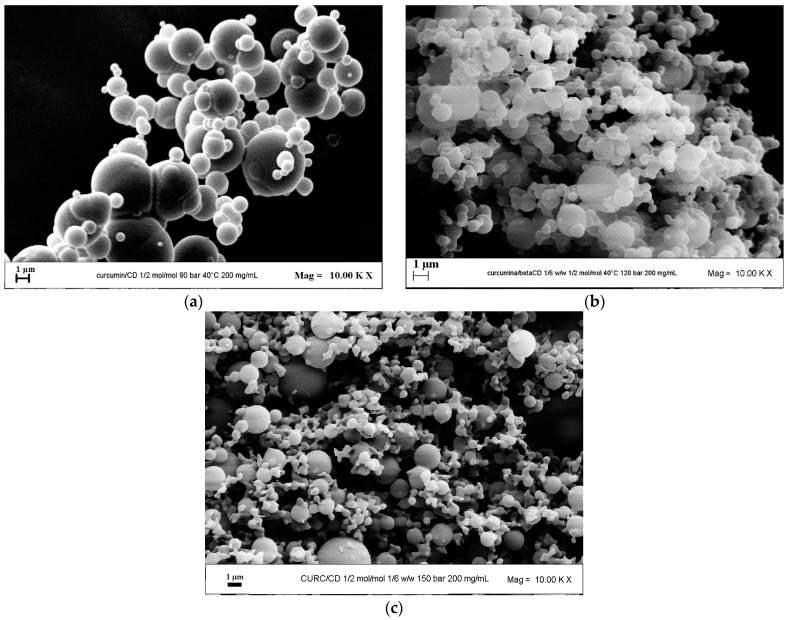
SAS powders of CURC/β-CD processed at 200 mg/mL and 1/2 mol/mol. FESEM images of the powders obtained at different pressures: (**a**) 9.0 MPa; (**b**) 12.0 MPa; (**c**) 15.0 MPa.

The effect of pressure was evaluated by fixing the operating pressure at 9.0, 12.0, and 15.0 MPa. The FESEM images in correspondence of the three pressures are reported in Figure 3. It is evident that when the operating pressure was increased, the mean diameter of the particles decreased, and the PSD narrowed. Since a bimodal size distribution at 15.0 MPa appears to have been obtained from the FESEM image reported in Figure 3c, revealing the presence of both small and larger particles, the particle size distribution was also determined by DLS with the Zetasizer tool. From the latter analysis, a mean particle size of 0.69 µm was found despite not obtaining a bimodal distribution.

The powders obtained by operating at different pressures are always in the micrometric range. However, considering that it is better, when possible, to operate at lower pressures for economic and environmental reasons, the effect of the API/carrier ratio was evaluated at 9.0 MPa in the subsequent set of experiments.

#### 3.1.3. Effect of CURC/β-CD Ratio

Once assessed that at 9.0 MPa and 200 mg/mL, well-defined microparticles are obtained using a CURC/β-CD molar ratio equal to 1/2, the micronization was attempted using an API/β-CD equimolar ratio (experiment #7 in Table 1). Indeed, it is well known from the literature that when cyclodextrin is used as a carrier, the obtained microparticles can consist of various units of complexes, and of the different guest/host configurations, the equimolar one is the most frequent [35,36]. Also, with a 1/1 mol/mol ratio, spherical particles in the micrometric range were obtained. As evident from the data reported in Table 1, when increasing the CURC/β-CD ratio, the mean particle size increased, and the particle size distribution enlarged. Considering that the powders coprecipitated at 9.0 MPa and 200 mg/mL at both ratios have the correct morphology and proper dimensions, the following analyses were performed on both. 

### 3.2. Experiments with PVP

#### 3.2.1. Effect of the Concentration

**Figure 4:**
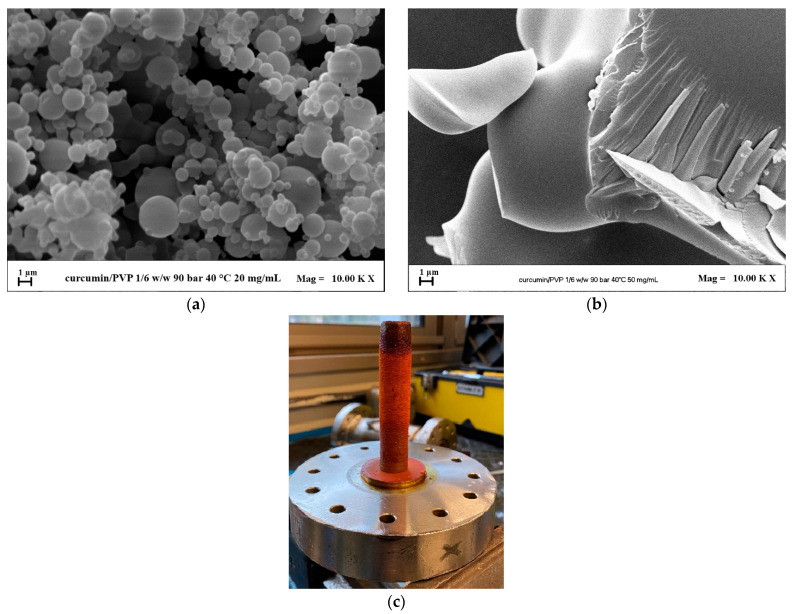
SAS powders of CURC/PVP processed at 9.0 MPa and 1/2 mol/mol. FESEM images at different concentrations: (**a**) 20 mg/mL; (**b**) 50 mg/mL. Photo of the filter after the test at 50 mg/mL (**c**).

The first set of tests using PVP as the carrier was conducted by varying C_TOT_ at P = 9.0 MPa and maintaining a CURC/polymer ratio equal to 1/2 mol/mol. A concentration of 20 mg/mL was chosen for the first test. Through the analysis by SEM microscopy of the samples, it was possible to verify the attainment of distinct particles whose average diameter is equal to 2.29 μm. The FESEM image of these particles is reported in Figure 4a. In the second test, a more concentrated solution at 50 mg/mL was injected, keeping the other operating conditions unchanged. At the end of the test, a moist powder with liquid was detected on the filter when the operating chamber was opened. In Figure 4b, a FESEM image that revealed the presence of crystals was reported, whereas, in Figure 4c, the filter’s picture after the precipitator’s opening showed the presence of a wet powder. It is therefore deduced that the increase in the concentration of the solutes at these operating conditions led to a shift of the mixture critical point toward higher pressure values. Therefore, the operating point fell within the miscibility hole. Considering that a concentration of 50 mg/mL led to an undesired morphology, the concentration was no further increased, and the following experiments were conducted at a concentration of 20 mg/mL. 

#### 3.2.2. Effect of the Operating Pressure

By setting a curcumin/PVP ratio equal to 1/2 mol/mol and a total solute concentration equal to 20 mg/mL, the pressure was increased from 9.0 MPa to 12.0 MPa.

**Figure 5:**
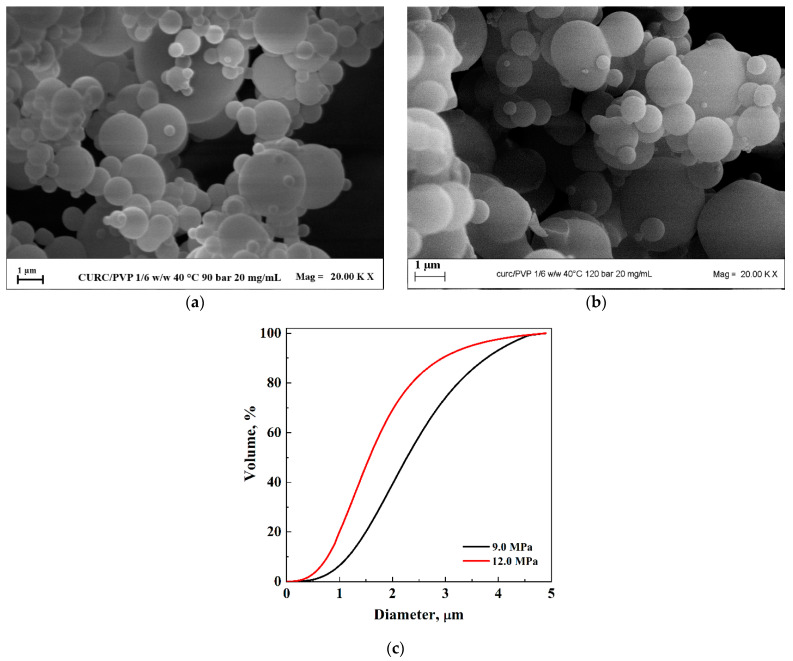
SAS powders of CURC/PVP processed at 20 mg/mL and 1/2 mol/mol. FESEM images with the pressure effect: (**a**) 9.0 MPa; (**b**) 12.0 MPa. Comparison of the cumulative volumetric PSDs (**c**).

The results obtained at a pressure of 9.0 MPa showed the obtaining of microparticles, as already discussed in the previous paragraph (the corresponding FESEM images are reported in Figure 4a and Figure 5a at different enlargements). The second test was conducted at 12.0 MPa, keeping all other operating conditions fixed. Also, in this case, microparticles were obtained (Figure 5b) whose mean diameter was equal to 1.72 μm. It is possible to observe from the comparison of the cumulative volumetric distributions reported in Figure 5c that when the pressure increased, the average diameter of the particles decreased. Since the distributions were wide, the curves were compared with those obtained by DLS analyses and were found to be superimposable.

#### 3.2.3. Effect of CURC/PVP Ratio

Once the previous parameters had been optimized (pressure of 12.0 MPa and concentration of 20 mg/mL), the effect of the CURC/PVP ratio was investigated. In this case, two ratios were considered: 1/2 mol/mol and 1/1 mol/mol. 

The first experiment of this set, conducted at a CURC/PVP ratio equal to 1/2 mol/mol, allowed the precipitation of particularly distinct spherical microparticles, which FESEM images and particle size distribution curve are reported in the previous sub-sections. Another test was conducted at a curcumin/PVP ratio equal to 1/1 mol/mol to obtain powders with a more active principle. In this case, nanometric or, at most, sub-micrometric particles were obtained, with an average diameter of 240 nm calculated by image analysis and approximately 264 nm by DLS.

### 3.3. Analyses

#### 3.3.1. FT-IR Analysis

Fourier transform spectroscopic analysis (FT-IR) was carried out to verify the presence of interactions between the functional group of curcumin and the carriers.

**Figure 6:**
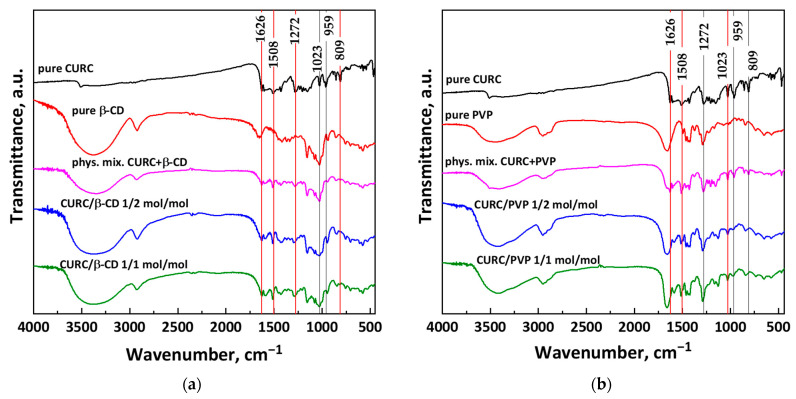
Comparison between the FT-IR traces of CURC-based powders: (**a**) inclusion complexes with β-CD and (**b**) coprecipitated powders with PVP.

As can be seen from Figure 6a, the FT-IR spectrum of β-CD displays a broad absorption band between 3571 cm^−1^ and 3220 cm^−1^ that can be attributed to the –OH group stretching; a band at about 2928 cm^−1^, which corresponds to the stretching of the –CH_2_ bond; and three bands at 1633 cm^−1^, 1153 cm^−1^, and 1022 cm^−1^ due to the stretching of C=O of cyclic alcohol, primary alcohol, and glycosidic bond, respectively [37].

Curcumin spectrum is rich in absorption bands due to its various functional groups, as is possible to observe in Figure 6a,b. The following characteristic vibrational bands were identified: stretching vibration of the C=O group at 1626 cm^−1^, stretching vibrations of ν(C=O) at 1508 cm^−1^, stretching vibration of enol C–O at 1272 cm^−1^, C–O–C stretching at 1023 cm^−1^, benzoate trans-CH vibration at 959 cm^−1^, and alkene CH in-plane bending at 809 cm^−1^ [37,38].

PVP spectrum reported in Figure 6b shows a broad absorption band at 3434 cm^−1^, which indicates the vibrational stretching of the –OH group; a peak at 2955 cm^−1^, indicating the vibrational stretching of the C–H bond; a peak at 1653 cm^−1^, indicating vibrational stretching of the C=O double bond; the absorption bands relating to the CN group, for the wavenumbers equal to 1496 and 1463 cm^−1^; and the absorption bands at 1440 and 1291 cm^−1^, which reveal the presence of CH groups [39].

Observing the traces related to the physical mixture and the SAS processed powders reported in Figure 6a, it is clear that the bands of β-CD dominate the spectra of CURC/β-CD powders; the latter do not have most of the characteristic bands of curcumin. However, some peaks linked to the active compound are clear (red vertical lines), a symptom that curcumin forms partial inclusion complexes with β-CD. The disappearance of most of the characteristic bands of curcumin is a sign of the formation of the inclusion complex, as the host molecule (β-CD) is located outside the complex formed and, thus, it hides the characteristic bands of the guest molecule at least partially. In Figure 6b, the spectra for curcumin and PVP are shown. Here again, the spectra of the physical mixture and the processed SAS samples have some characteristic bands of curcumin (indicated with red vertical lines). However, there is a greater correspondence with the spectrum of the polymer, as it is present more in the powders.

#### 3.3.2. Dissolution Tests

**Figure 7:**
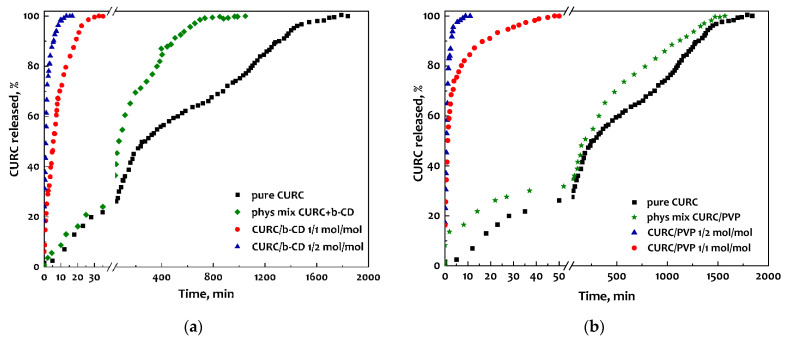
Comparison between the dissolution tests of CURC-based powders: (**a**) inclusion complexes with β-CD and (**b**) coprecipitated powders with PVP.

UV-vis spectroscopy was used to compare the dissolution of CURC in pure and coprecipitated form with the two different carriers. The CURC/β-CD system was first analyzed. In Figure 7a, it is possible to observe the comparison of the release of unprocessed curcumin, of the curcumin/β-CD physical mixture, and of the inclusion complexes at different curcumin/carrier ratios (1/2 mol/mol and 1/1 mol/mol).

Unprocessed CURC dissolved entirely after about 33 h, while the physical mixture took about 17 h. In the case of the powders obtained by coprecipitating curcumin with β-cyclodextrin, it is clear that, in both cases analyzed, the release of the antioxidant was significantly speeded up. In particular, for the curcumin/β-CD molar ratio equal to 1/2 mol/mol, the complete dissolution of the active ingredient was observed in approximately 15 min, while with a higher molar ratio (1/1 mol/mol), the complete dissolution in PBS occurred in approximately 30 min. It is therefore clear that not only the presence of β-CD but also the intimate contact between it and CURC inside the microparticles allows the dissolution speed of the active compound to be substantially increased.

Analyzing the release kinetics of the physical CURC/PVP mixture and the CURC/PVP coprecipitated powders (reported in Figure 7b), it is possible to observe, in the first case, a very similar trend to that of unprocessed curcumin, with complete dissolution in solution after approximately 25 h. In the case of coprecipitated powders, curcumin is released very quickly. In particular, considering a lower mass ratio (1/2 mol/mol), complete dissolution occurs after approximately 10 min, while for the sample processed at a higher mass ratio (1/1 mol/mol), complete dissolution occurs in approximately 45 min.

#### 3.3.3. Stoichiometry of Inclusion Complexes

**Figure 8:**
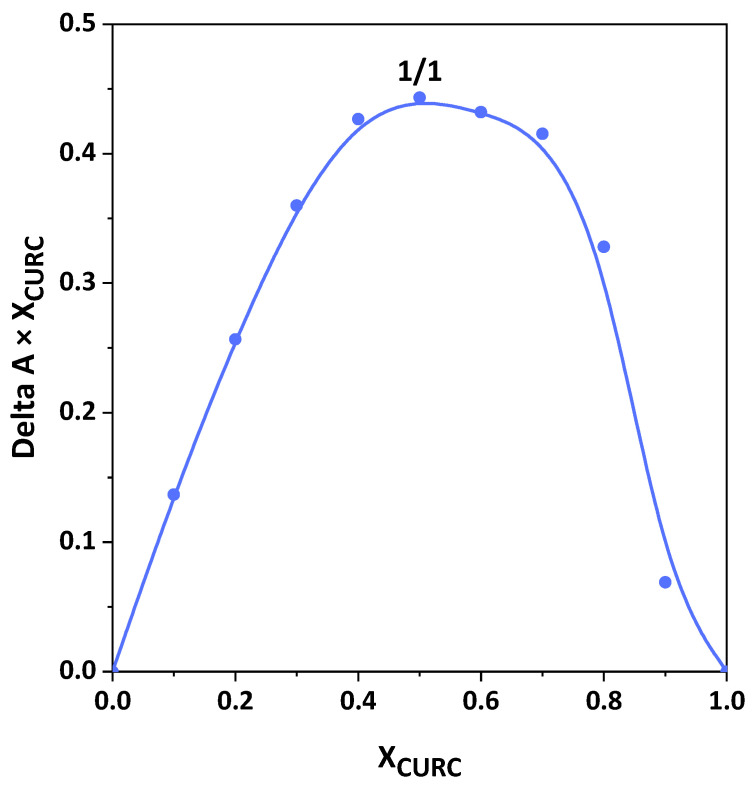
Evaluation of the stoichiometry of CURC/β-CD complexes through Job plot.

The stoichiometric ratio of guest to host molecules was determined using the Job plot, reported in Figure 8. The maximum was found at X = 0.5, corresponding to the equimolar ratio. This analysis defines the best stoichiometric ratio for forming a stable inclusion complex between CURC and β-CD.

## 4. Conclusions

Based on the tests and characterization performed, it can be said that the SAS method is effective for both the complexation and coprecipitation of curcumin. The production of β-CD-based complexes or PVP-based coprecipitates can accelerate the dissolution of the active ingredients studied and thus achieve the established goals. However, the use of β-CD appears more advantageous than PVP as it reduces the amount of carrier in the composite powder while ensuring rapid release, thereby improving bioavailability. Ultimately, this research makes it possible to produce inclusion complexes capable of increasing the therapeutic effects of antioxidants such as curcumin, which can be used to prevent or treat many diseases related to cellular oxidative stress.

## Figures and Tables

**Table 1:** SAS experiments performed to obtain inclusion complexes and coprecipitated particles (AGG = agglomerates; C = crystals; MP = microparticles; cMP = coalescing microparticles).

#	Carrier	CURC/Carrier (mol/mol)	P (MPa)	C_tot_ (mg/mL)	Morphology	m.d. ± s.d. (μm)	EE%
1	-	-	9	20	-	-	-
2	β-CD	1/2	9	100	AGG	-	62.0
3				150	C + MP	-	70.3
4				200	MP	2.98 ± 0.92	99.4
5			12	200	MP + cMP	1.43 ± 0.46	61.8
6			15	200	MP + cMP	1.35 ± 0.48	59.6
7		1/1	9	200	MP	3.69 ± 1.24	71.7
8	PVP	1/2	9	20	MP	2.29 ± 0.78	82.9
9				50	C	-	-
10			12	20	MP	1.72 ± 0.56	99.6
11		1/1	12	20	cMP	0.24 ± 0.10	99.6

## Data Availability

Data are contained within the article.
